# An Antarctic lichen isolate (*Cladonia borealis*) genome reveals potential adaptation to extreme environments

**DOI:** 10.1038/s41598-024-51895-x

**Published:** 2024-01-16

**Authors:** Minjoo Cho, Seung Jae Lee, Eunkyung Choi, Jinmu Kim, Soyun Choi, Jun Hyuck Lee, Hyun Park

**Affiliations:** 1 Department of Biotechnology, College of Life Sciences and Biotechnology, Korea University, Seoul, 02841 South Korea; 2 Research Unit of Cryogenic Novel Material, Korea Polar Research Institute, Incheon, 21990 South Korea; 3 Department of Polar Sciences, University of Science and Technology, Incheon, 21990 South Korea

**Keywords:** Fungi, Fungal genomics

## Abstract

*Cladonia borealis* is a lichen that inhabits Antarctica’s harsh environment. We sequenced the whole genome of a *C. borealis* culture isolated from a specimen collected in Antarctica using long-read sequencing technology to identify specific genetic elements related to its potential environmental adaptation. The final genome assembly produced 48 scaffolds, the longest being 2.2 Mbp, a 1.6 Mbp N50 contig length, and a 36 Mbp total length. A total of 10,749 protein-coding genes were annotated, containing 33 biosynthetic gene clusters and 102 carbohydrate-active enzymes. A comparative genomics analysis was conducted on six *Cladonia* species, and the genome of *C. borealis* exhibited 45 expanded and 50 contracted gene families. We identified that *C. borealis* has more *Copia* transposable elements and expanded transporters (ABC transporters and magnesium transporters) compared to other *Cladonia* species. Our results suggest that these differences contribute to *C. borealis*’ remarkable adaptability in the Antarctic environment. This study also provides a useful resource for the genomic analysis of lichens and genetic insights into the survival of species isolated from Antarctica.

## Introduction

Lichens are composite organisms that exist in a symbiotic association between lichen-forming fungi (LFF, mycobiont) and partner algae or cyanobacteria (photobiont)^1,2^. This fungal lifestyle constitutes approximately 20% of all known fungal species^2^. The photobiont provides sustenance to the mycobiont via the fixation of carbohydrates and atmospheric nitrogen while sheltering under the protection of the LFF^2^. The lichen symbiosis is an interesting paradigm for the study of environmental adaptation. Lichens exhibit the capacity to endure in extreme environments, such as the cryogenic, arid, and high solar radiation conditions present in Antarctica^3^. However, lichen’s astonishing environmental adaptation abilities are still not fully understood, and numerous studies are being conducted to study lichen's ability to adapt to the environment.

Lichens are one of the dominant autotrophs on the Antarctic Peninsula^4^. Antarctica and South Georgia Island are home to a diverse range of lichens, accommodating approximately 427 taxa^5^. Within the Antarctic vegetation, *Cladonia borealis* stands out as one of the dominant organisms^1^. *Cladonia borealis*, which belongs to the class Lecanoromycetes in the Ascomycota division of the Fungi kingdom, is found mainly in the soil, humus, and mosses of polar, subpolar, and alpine areas^6^. Although limited data is available for the growth rate of lichens in continental Antarctica, there is a general consensus that their growth is comparatively slower than that of lichens in other global regions^7^. Research efforts of *C. borealis* can contribute to advancing our understanding of the ecological impacts of lichen on the Antarctic region.

The ecological plasticity and evolutionary mechanisms that enable lichens to persist in various environments may be elucidated through the scrutiny of biological data, including genomic analyses. Consequently, advanced sequencing technologies have the potential to aid in unraveling these mysteries. For example, studies have shown that viruses can play an important role in symbioses, increasing the association’s adaptability to the environment^8^. Diverse genetic elements are analyzed through genomic assembly and annotation process. Genetic elements have important roles in the survival of organisms. For instance, transposable elements (TEs) constitute a major part of any genome and play important roles in gene expression, genomic rearrangements, epigenetic variation, mutations, and phenotypic variation^9^. Most TEs remain quiescent and are activated by environmental cues. Lichens are an attractive source to study TE divergence, but not enough studies have been done.

Lichens are also known as putative natural product producers. Using bioinformatic data, diverse analyses to reveal lichen’s secondary metabolites have been conducted. These metabolites have proven distinctive and bioactive, and over 1000 such compounds have been identified lichen exclusive to date^10^. Some exhibit pharmacological activities, including antibacterial, antifungal, and anti-inflammatory effects^11^. The lichen secondary metabolites atranorin and physciosporin have been shown to possess inhibitory properties against lung cancer cell activity^12,13^. Furthermore, some natural pigments derived from fungi are known to contribute to environmental adaptation and abiotic stress tolerance^14^. These examples suggest that lichen can produce compounds that can advance pharmaceutical science and ecology. Through genetic studies, we can discover biosynthetic gene clusters (BGCs) with high possibilities of producing these compounds, such as polyketide synthase (PKS), nonribosomal peptide synthetase (NRPS), terpene, and hybrid PKS-NRPS types of natural products^10^.

Fungal carbohydrate-active enzymes (CAZymes) are enzymes which can catalyze glycans and glycoconjugates. Fungi produce an array of CAZymes for utilization of substrates. Fungal carbohydrate-active enzymes can be classified into six main classes, glycoside hydrolases (GHs), Carbohydrate esterases (CEs), polysaccharide lyases (PLs), glycosyl transferases (GTs), auxiliary activities (AAs), and carbohydrate binding modules (CBMs). For processing carbohydrates, fungi mostly rely on GHs, GTs, and AAs^15^. In addition to the catalytic modules, CBMs are common in enzymes active in cell-wall hydrolysis^16^. CAZyme families play important roles in lignocellulose breakdown, simple sugar metabolism, biofilm formation, antimicrobial biosynthesis, and diverse nutrient cycling routes^17^. LFFs absorb and utilize nutrients from phototrophic organisms and are thus classified as composite autotrophs. CAZymes represent an intriguing target for investigation in the genome of LFF, as mycobionts do not directly absorb nutrients from organic matter. Resl et al. have substantiated that CAZyme loss did not occur in LFF^18^. Their result revealed intricate patterns of retention and loss, which are not consistently aligned with the notion of CAZyme erosion following phototrophic symbiotic acquisition.

Transporter proteins are essential types of transmembrane proteins, their function is important in cell nutrition, communication, stress resistance, and homeostasis. One of the largest transport protein superfamilies in plants and fungi, ABC transporters, which are membrane components, are vital for cell energy regulation, a function vital for cell survival^19^. In lichen genomes, a wide range of transporter proteins is present, similar to other organisms. Notably, lichen forms a symbiotic relationship with partner algae, underscoring the increased significance of the transport system in these organisms.

In this study, we performed whole genome sequencing of *C. borealis* using PacBio single-molecule real-time (SMRT) sequencing, which has been widely used for de novo assembly^20^. A comparative analysis using genomes of the genus *Cladonia* was conducted. Secondary metabolite BGCs, CAZymes, and putative environmental adaptation related genes were also analyzed. This study provides a basic resource for the whole-genome sequencing of lichen and genetic insights into Antarctic lichen isolate.

## Results

### Genome assembly of *C. borealis*

**Figure 1:**
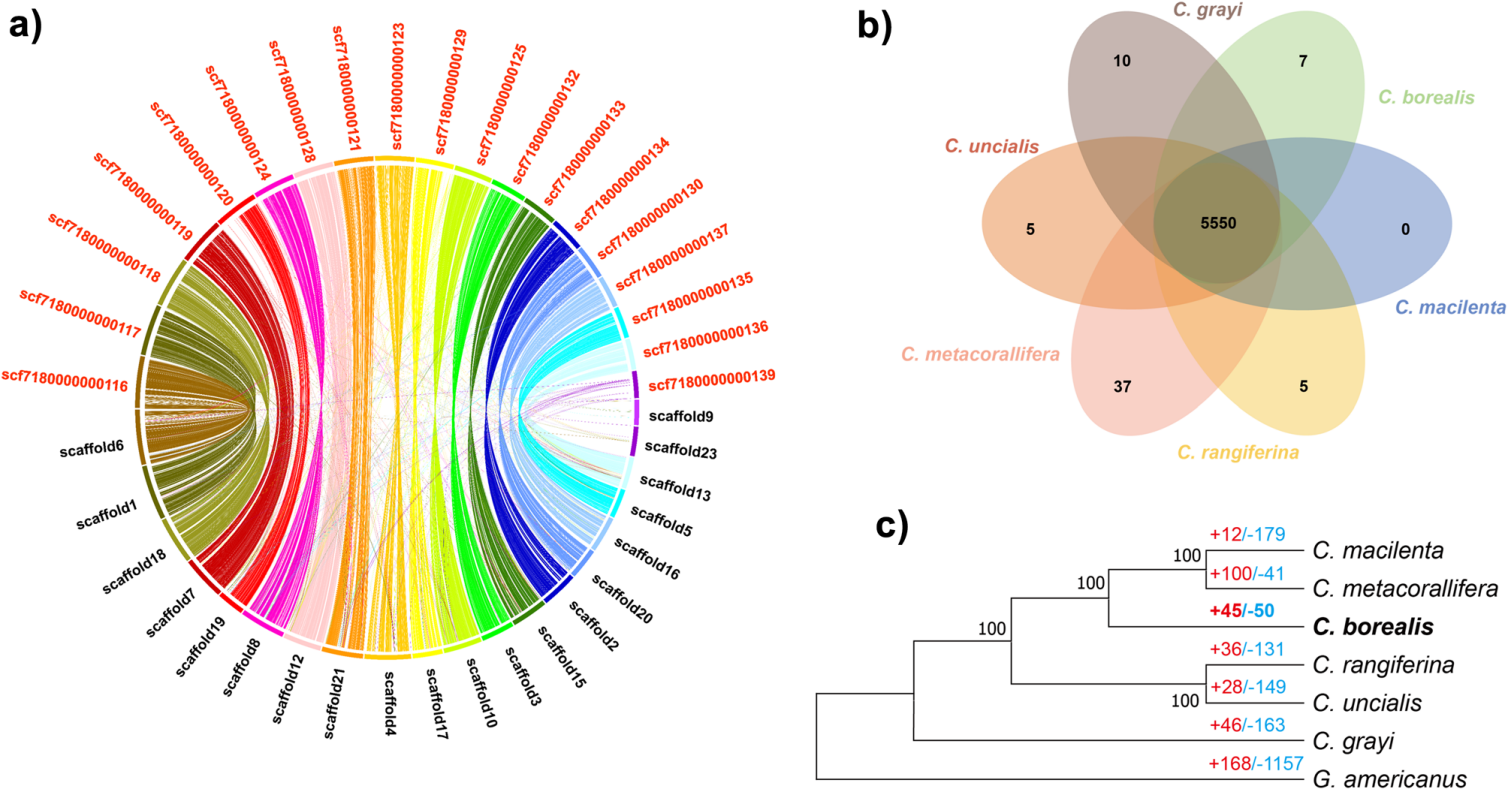
Genome synteny and comparisons between six *Cladonia* species. (**a**) Circos plot showing conservation of synteny between *C. borealis* and *C. metacorallifera*. The synteny map represents 20 *C. metacorallifera* scaffolds (black) and 19 *C. borealis* scaffolds (red). (**b**) Venn diagram, created using the Orthovenn 2 web platform, showing the numbers of gene families shared among *Cladonia* species and unique to each species. (**c**) Maximum-likely hood tree, created using MegaX, based on the sequences of single-copy orthologous proteins The numbers of gene families that have expanded (red, +) and contracted (blue, −), as inferred using CAFÉ, are represented.

PacBio SMRT sequencing produced 635,943 long reads and 4.9 Gbp data. Reads over 10 Kbp accounted for more than 58% of the total reads (Table S1). Through de novo assembly, we produced 48 scaffolds, the longest being 2.2 Mbp, with an N50 scaffold length of 1.6 Mbp and a 36 Mbp total scaffolds length after the polishing process (Table 1). Assembly completeness was confirmed with Benchmarking Universal Single-Copy Orthologs (BUSCO), among 1706 Ascomycota orthologous genes, 1601 (93.9%) complete genes were identified. Of these complete BUSCOs, 1595 (93.5%) and six (0.4%) genes were single-copy and duplicated BUSCOs, respectively. As fragmented and missing BUSCOs, 18 (1.1%) and 87 (5.0%) genes were confirmed (Table 2). The genome synteny between *C. borealis* and *C. metacorallifera* showed a high level of conservation (Fig. 1a).

**Table 1:** Assembly and annotation information for the *C. borealis* genome*.*

Assembly	
Number	48
Total size (bp)	36,013,978
Longest scaffolds (bp)	2,231,325
N50 scaffold length (bp)	1,657,173
Number of scaffolds > 1 Mb	19
G + C content (%)	45.1

Annotation database	Annotated no.
No. of genes	10,749
Pfam	3930 (36.6%)
SignalP	9703 (90.3%)
TmHMM	1966 (18.3%)
Swissprot blastx	6280 (58.4%)
Swissprot blastp	6437 (59.9%)

**Table 2:** Assembly completeness of the *C. borealis* genome, estimated using BUSCO v4.1.2.

Ascomycota_odb10	No.
Complete BUSCOs (C)	1601 (93.9%)
Complete and single-copy BUSCOs (S)	1595 (93.5%)
Complete and duplicated BUSCOs (D)	6 (0.4%)
Fragmented BUSCOs (F)	18 (1.1%)
Missing BUSCOs (M)	87 (5.0%)
Total BUSCO groups searched	1706

### Genome annotation

A total of 11,677 repeat elements were identified, which accounted for 10.34% of the total *C. borealis* genome. Repeat sequences included long terminal repeats (LTRs, 5.91%), long interspersed nuclear elements (LINEs, 0.01%), and DNA transposons (0.43%) (Table S2).

Through EVidenceModeler annotation, 10,749 protein-coding genes were annotated. The total length of exons was 17 Mbp, with an average number of 3.11 exons per gene. For the functional annotation, 3930 (36.6%), 9703 (90.3%), 1966 (18.3%), 6280 (58.4%), and 6437 (59.9%) genes were annotated in Pfam, SignalP, TmHMM, Swissprot blastx, and Swissprot blastp, respectively (Table 1). The distribution of the top 20 gene ontology (GO) terms in level 5 is shown in Fig. S1. Among them, 1280 genes were annotated as intracellular membrane-bounded organelle (GO:0043231) related genes, making this the most abundant GO term in the *C. borealis* genome.

### Comparative genomics

The genome completeness of all *Cladonia* species (C. *macilenta, C. metacorallifera, C. rangiferina, C. uncialis*, and *C. grayi*) and *Gomphillus americanus* were identified to be above 90%, and the number of annotated genes ranged from 8200 to 10,825 (Table 3, Table S3). We classified repeat elements based on superfamily and found that the *C. borealis* genome contained the greatest total lengths of Kolobok-H and PIF-Harbinger types of DNA transposons and simple repeats. Also, regarding types of LTR transposable elements (TEs), *Copia*, ERVK, and Pao types of LTR TEs were the most common types in the *C. borealis* genome (Table S2).

**Table 3:** Species and information for the genomes used in this study.

Organisms	Abbr.	Accession No.	Project no.	Sampling location
*Cladonia borealis*	Cbo	GCA_018257855.1	PRJNA693578	King George Island, Antarctica
*Cladonia grayi*	Cgr	Cgr/DA2myc/ss v2.0	PRJNA731936	North Carolina, USA
*Cladonia macilenta*	Cma	GCA_000444155.1	PRJNA210603	Mt. Cangshan, China
*Cladonia metacorallifera*	Cme	GCA_000482085.2	PRJNA219240	Mt. Seorak, South Korea
*Cladonia rangiferina*	Crg	GCA_006146055.1	PRJNA418168	Deciduous woodland, USA
*Cladonia uncialis*	Cuc	GCA_002927785.1	PRJNA348097	Manitoba, Canada
*Gomphillus americanus*	Gam	GCA_905337335.1	PRJEB42325	Granitic flatrock border woodland, USA

*C. grayi* was downloaded from JGI and other species were obtained from NCBI.

### Prediction of BGCs and CAZymes

**Figure 2:**
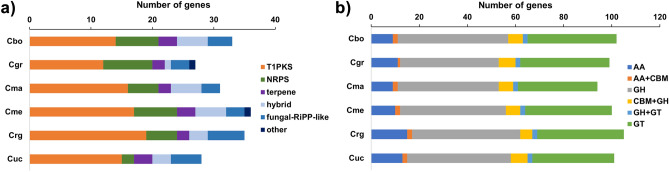
Secondary metabolite synthase gene clusters and CAZyme genes found in the six *Cladonia* species’ genomes. (**a**) Secondary metabolite clusters, including type I polyketide synthases (T1PKS), non-ribosomal peptide synthetases (NRPS), terpenes, hybrid, fungal-RiPP-like, and other clusters, in the *Cladonia* genomes. (**b**) Number of CAZyme genes, including auxiliary activities (AA), glycoside hydrolases (GH), and glycosyl transferases (GT) and combined two-gene domains comprising GH and GT (GH + GT) or carbohydrate-binding modules (CBM) and AA(AA + CBM) or GH (CBM + GH), in the *Cladonia* genomes. See Table 3 for species abbreviations.

In total, 33, 27, 31, 36, 35, and 28 BGCs were identified in *C. borealis*, *C. grayi*, *C. macilenta*, *C. metacorallifera*, *C. rangiferina*, and *C. uncialis* genomes, respectively. Fourteen type I PKS (T1PKS), seven NRPS, three terpenes, five hybrid clusters, and four fungal ribosomally synthesized and posttranslationally modified peptide product (RiPP)-like clusters were involved in the secondary metabolism of *C. borealis* (Fig. 2a). Among the 33 gene clusters of *C. borealis*, 14 were matched with similar known clusters. Six predicted natural compounds were found only in *C. borealis* genome: CJ-16,173/CJ-15,696/citridone B/citridone A/citridone B’, pseurotin/azaspirene, secalonic acids, xenoacremone A, 6-hydroxymellein, and wortmanamide A/wortmanamide B with similarity score 12% to 83%.

We identified 102, 99, 94, 100, 105, and 101 CAZymes in *C. borealis*, *C. grayi*, *C. macilenta*, *C. metacorallifera*, *C. rangiferina*, and *C. uncialis* genome, respectively. In *C. borealis*, CAZymes included 11 auxiliary activities (AA) distributed in two families, AA1 and AA3; 45 glycoside hydrolases (GH) in 22 families; and 81 glycosyl transferases (GT) in 19 families. Additionally, enzymes containing two domains were detected, two AA/carbohydrate binding modules (CBM), six GH/CBM, and two GH/GT (Fig. 2b, Table S6).

### Expanded genes in *C. borealis*

In the six *Cladonia* mycobionts, a total of 5550 gene families were shared and seven gene families were identified as *C. borealis* specific. Seven gene families were annotated with GO terms (GO:0055085, transmembrane transport; GO:0004674, protein serine/threonine kinase activity; GO:0016705, oxidoreductase activity, acting on paired donors, with incorporation or reduction of molecular oxygen; and 4 not represented in the GO database) (Fig. 1b).

**Figure 3:**
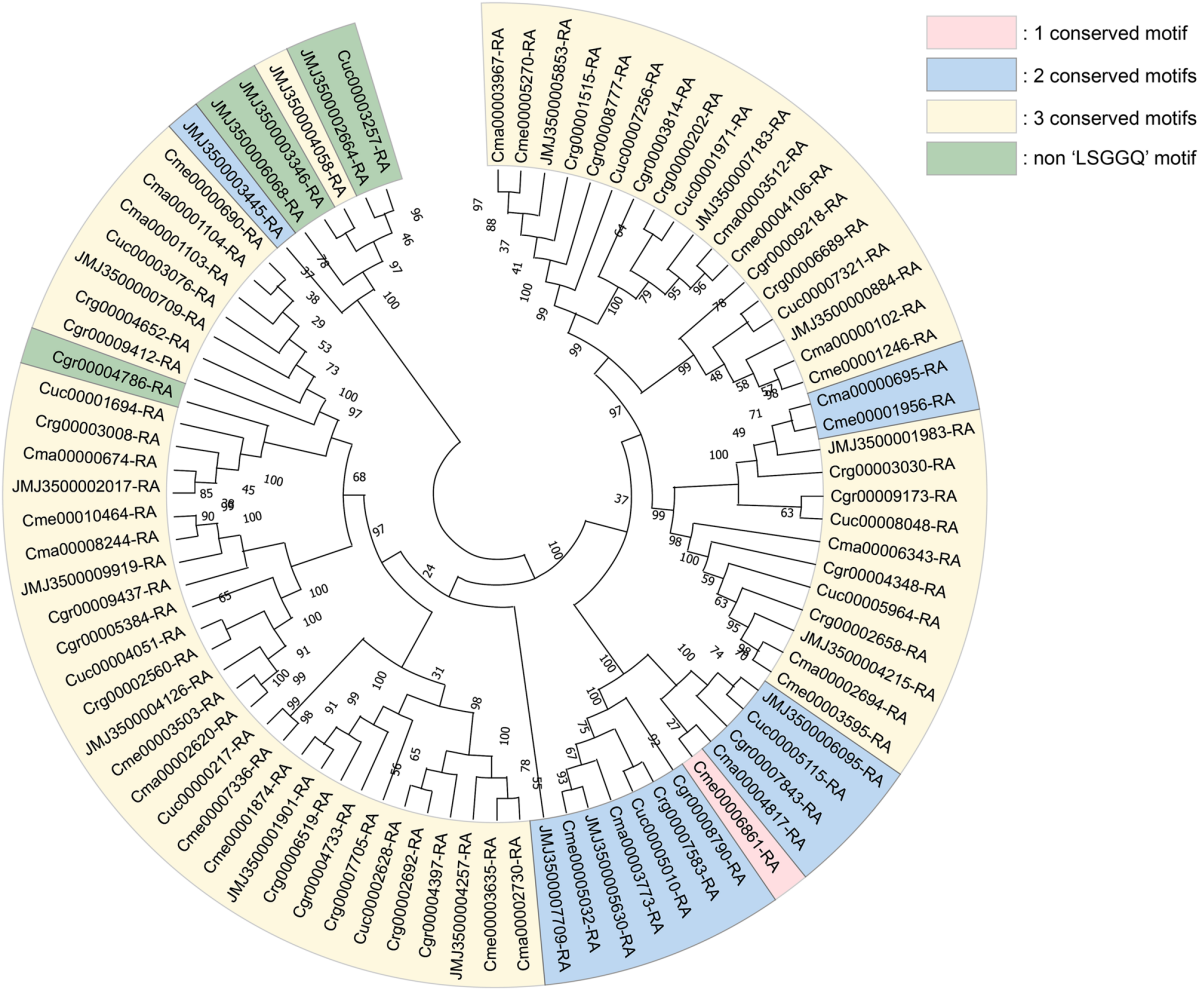
Phylogenetic tree depicting the relationships among the ABC transporter proteins of the six *Cladonia* species. Most genes possessed three conserved motifs, while there are 1 and 14 genes with one and two motifs, respectively. Five genes did not possess the “LSGGQ” motif, with three genes belonging to *C. borealis* and the other two genes originating from *C. grayi* and *C. uncialis.* Each protein identifier contains the species abbreviation (see Table 3); “JMJ” represents *C. borealis*.

**Figure 4:**
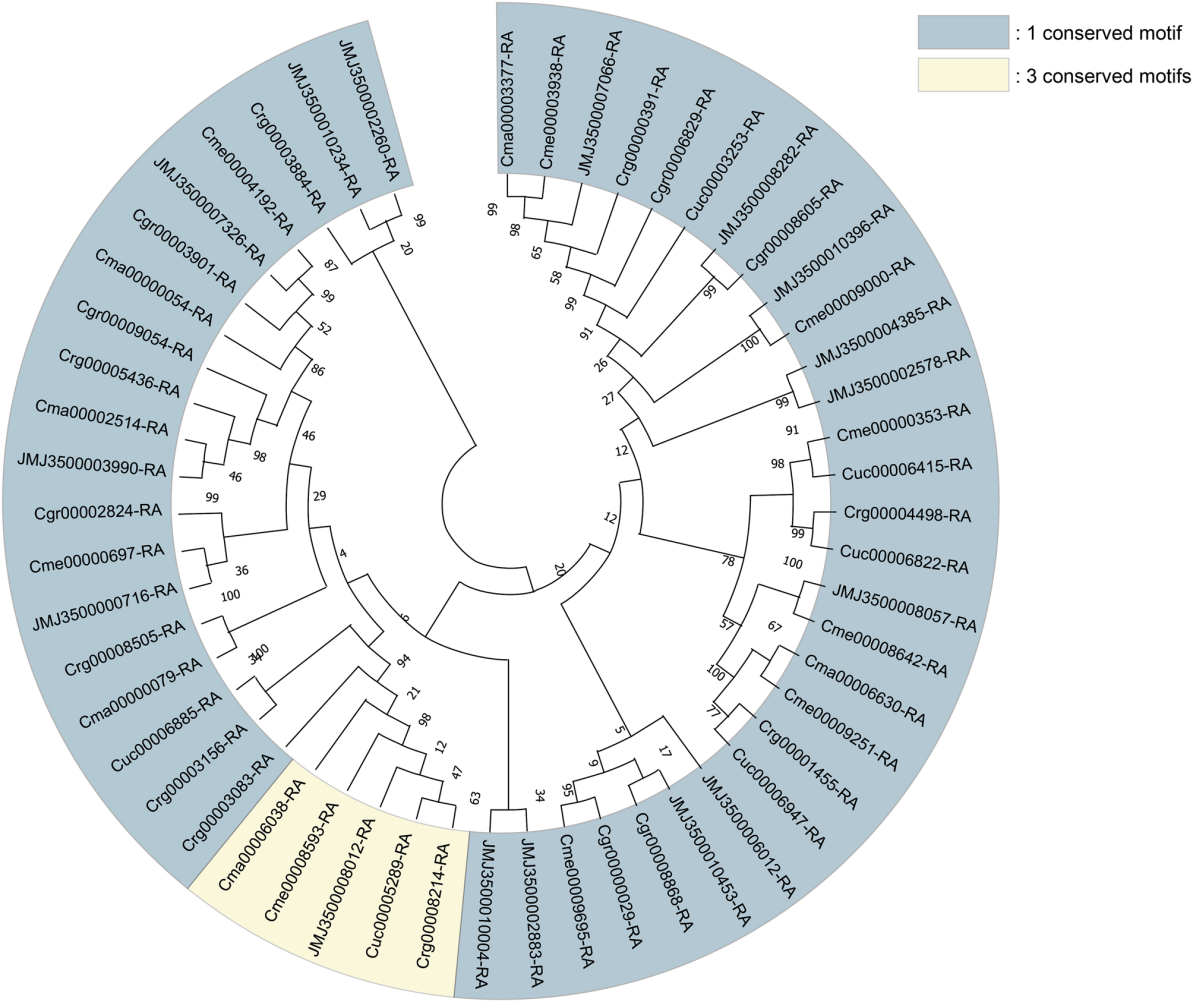
Phylogenetic tree depicting the relationships among the magnesium transporter proteins of the six *Cladonia* species. Except for five, all genes contained one conserved domain. These five exhibited three domains, with *C. grayi* being the only species not containing any genes with the three conserved motifs. Each protein identifier contains the species abbreviation (see Table 3); “JMJ” represents *C. borealis*.

The *C. borealis* genome showed expansion in 45 and contraction in 50 gene families (Fig. 1c). For the expanded gene families, Fisher’s exact tests were used to identify enriched GO terms using Omicsbox. The 20 most significantly enriched GO terms of *C. borealis* are shown in Fig. S2. Transporter-related GO terms were the most enriched annotation in expanded gene families (Fig. S2). We manually annotated transporter-related proteins, and genes were classified according to their function. *Cladonia borealis* had the highest number of ATP-binding cassette (ABC) transporters and magnesium transporters (MGTs) among the studied species with 19 ABC transporter proteins and 16 MGTs (Table 4). The ABC transporters were located in 12 scaffolds, while MGTs were located in 10 (Tables S4, S5). Most of the ABC transporter proteins included a signature motif, LSGGQ, which participates in nucleotide binding. Only five protein sequences did not contain this motif, JMJ3500003346-RA, JMJ3500002664-RA, JMJ3500006068-RA, Cgr00004786-RA, and Cuc00003257-RA (Fig. 3)^21,22^. Most of the MGTs revealed one conserved motif, but five sequences—JMJ3500008012-RA, Cma00006038-RA, Cme00008593-RA, Crg00008214-RA, and Cuc00005289-RA—included three conserved motifs (Fig. 4).

**Table 4:** Number of transporter proteins in each *Cladonia* species.

Type of transporter proteins	*C. borealis*	*C. grayi*	*C. macilenta*	*C. metacorallifera*	*C. rangiferina*	*C. uncialis*
ABC transporter	19	13	14	13	12	13
Magnesium transporter	16	7	6	9	9	6

## Discussion

As genome sequencing technology has become accessible, genome-level studies of living organisms have become widely used to explore diversity. Using genomic data, much effort has focused on revealing environmental adaptation in a diverse array of organisms. Lichens are notable for their ability to survive in extreme abiotic stress, but more studies using genome data are needed to clarify environmental adaptation mechanisms in lichens. In this study, we assembled and annotated the genome of *C. borealis* using PacBio SMRT sequencing data to identify potential adaptation behind this Antarctic native lichen. We performed comparative genomics analyses among different *Cladonia* species using a variety of bioinformatic tools to identify genes specific to *C. borealis*. The *Cladonia* species utilized in this study were selected for the purpose of comparing lichens isolated from Antarctic regions with those isolated from temperate climate environments.

Repeat elements represented 5.73 to 12.76% of the total genomes of the six *Cladonia* species, and about one-third of the repeat elements in each genome were uncharacterized. Like typical fungal genomes, LTR *Gypsy* transposons were more common than *Copia* elements in every species^23^. While repeat elements occupy a large amount in the genomes of animals and plants, these results indicate that repeat elements represent a relatively small proportion in lichen, but so many repeat elements are still unknown^24^. The *C. borealis* genome contained the most diverse set of repeat element types among the six species. We observed that the LTR *Copia* superfamily was expanded in the *C. borealis* genome. The activation of retrotransposons in response to stressors and extreme change is common in eukaryotes^25–27^. In grasses, it has been observed that *Copia* transposable elements are more frequently inserted near genes associated with stress response^28^. Similarly, in pitaya plants and *Dendrobium officinale*, *Copia* retrotransposons exhibit transcriptional activation, and among environmental stressors, transcriptional activation was highest in response to cold stress in particular^29,30^. Previous research, along with the findings of this study, suggests that transposable elements play a role in lichen’s remarkable environmental adaptability, especially in the harsh Antarctic environment.

In many organisms, including lichen-forming fungi, secondary metabolites have putative roles in responses to environmental pressures^14^. Also, some terpenoid compounds are derived from plant metabolites and are known to protect plants from abiotic stressors or have diverse functions in the interactions between plants and their environment^31^. But our results showed that *C. borealis*, *C. metacorallifera*, and *C. uncialis* genome revealed same number of terpene gene clusters. We have verified the presence of six distinct natural compounds within the *C. borealis* genome. However, an assessment of their potential influence on Antarctic adaptation remains pending. While our research has been conducted to explore the genetic mechanisms underlying adaptation in an Antarctic lichen, thus far, no specific BGCs have been identified that are specifically associated with survival in Antarctica. Uncovering the potential genetic pathways involved in adaptation to this unique environment will require further exploration. Also, we compared six *Cladonia* species to characterize CAZyme divergence in each genome. The *C. borealis* genome possesses the largest numbers of GHs and GTs and diverse CAZyme families, but the differences between the *Cladonia* species were insignificant.

In symbiotic partner relationships, transporter proteins play a key role in nutrient and signal exchanges. We found transmembrane transporter protein types that are expanded in *C. borealis* through Computational Analysis of gene Family Evolution (CAFÉ), OrthoFinder, and Orthovenn 2 analyses, and with functional classification, we identified the specificity of ABC transporters and MGTs in the genome of *C. borealis*.

Previous studies have found that genomes of cold-tolerant strains of bacteria harbor increased numbers of transport proteins, including ABC transporters, and that ABC transporters play an important role in abiotic stress response including cold adaptation^32–35^. Fungal ABC transporters, in particular, have been shown to work as natural toxin or natural product efflux pumps^36^. Under extreme cold stress, such as is found in the Antarctic environment, organisms have difficulty in accessing nutrients and maintain cell homeostasis. Under such conditions, this class of transporters might perform key functions in maintaining the consistency of nutrient uptake and energy availability, important processes for lichen survival.

Our analysis has confirmed the presence of numerous MGTs in the *C. borealis* genome, which has directed our attention towards investigating the role of magnesium in the Antarctic environment. Lichens do not depend directly on the soil but rather utilize nutrients from the air^37^. On King George Island, Antarctica, insoluble airborne particulates in ice are composed of aluminum, potassium, magnesium, iron, and calcium. Furthermore, in Antarctic ice-rich sediments, extensive salt accumulation with major cations (Na^+^, Ca^2+^, K^+^, and Mg^2+^) occurs^38^. These findings suggest that the Antarctic atmosphere contains a significant amount of magnesium, and these aerosols may contribute to the accumulation of Mg^2+^ in the soil. These environmental conditions expose living organisms to extreme osmotic and abiotic stress. Osmotic homeostasis is significant for cell survival: under osmotic stress, cells remain viable by importing compatible solutes via membrane transport proteins to regulate osmotic pressure and maintain turgor. Magnesium efflux and import systems maintain magnesium homeostasis, which is critical for organisms to adapt to high-magnesium environments^39^. Thus, the function of the magnesium transporter becomes crucial in maintaining cellular homeostasis during osmotic stress and response to abiotic stress^40,41^. *Cladonia borealis* can live in the Mg^2+^-rich Antarctic environment, which suggests a requirement for more MGTs. Also, previous study has revealed that MGTs are upregulated under cold stress in *Yersinia pseudotuberculosis*^42^. *Cladonia borealis*’s significantly higher number of MGTs among the *Cladonia* species might represent potential adaptation to the environment of Antarctica. Our results provide evidence of putative cold environmental adaptation related genes in Antarctic dominant *C. borealis* through a comparative analysis with other *Cladonia* species isolated from temperate climate.

## Conclusion

Here, we report the genome of *C. borealis*, based on PacBio RSII long-read DNA sequencing data. The genes specific to the Antarctic-derived lichens can be identified through comparison between Antarctic and temperate species isolates. In the genome of *C. borealis*, we have identified *Copia* transposable elements and an expanded set of ABC transporters and MGTs that potentially play helpful roles in enduring the extreme Antarctic environment. Transposable elements are known to be involved in significant genetic phenomena, and the expanded *Copia* TEs could potentially provide support for the survival of living organisms under extreme environmental stress. Additionally, the expanded ABC transporters and magnesium transporters may play roles in maintaining cellular homeostasis in *C. borealis* compared to species from temperate climates. This study should serve as a useful resource for the genomic analysis of lichens, contributing to the advancement of understanding and harnessing the genetic resources within this group of organisms. The genome sequencing data of *C. borealis* has revealed information on genes potentially related to environmental adaptation in the Antarctic and has contributed to advancing understanding of an Antarctic dominant lichen mycobiont.

## Methods

### Sample preparation and genome sequencing

The *Cladonia borealis* specimen was collected from King George Island, Antarctica (S 61° 59′ 17′′, W 58° 1′ 10′′). We removed contaminants and isolated the *C. borealis* mycobiont. The isolated specimen was pure cultured in the laboratory for PacBio sequencing. The fungal isolate was grown under dark conditions at 15 °C on malt-yeast (MY) agar medium (15 g malt extract, 15 g/L agar). The lichen voucher material is stored in the ‘Korean Lichen & Allied Bioresource Center’ (https://cc.aris.re.kr/kolabic/app/main/mainView.do) with resource number 022262. Mycobionts’ genomic DNA was extracted using a method previously described by Varela-Alvarez et al.^43^, with some modifications. The 400 mg of tissue from *C. borealis* was homogenized with liquid nitrogen in the mortar. Then, 8 mL of lysis buffer (50 mM Tris-HCl pH 8.0, 200 mM NaCl, 20 mM EDTA, 2% SDS, Proteinase K 20 mg/mL) was added and the sample was mixed. Next 2.4 mL of pre-heated CTAB buffer was added and the sample was heated at 65 °C for 1 h. One volume of phenol-chloroform-isoamyl alcohol (25:24:1) was added to the sample and mixed by inversion 10 times and then centrifuged for 10 min at 13,200 rpm. The aqueous phase was collected into a clean microcentrifuge tube and the rest was discarded. Two volumes of absolute ethanol were added and mixed gently. The sample was left for at least 30 min at − 20 °C and afterwards centrifuged for 10 min at 13,200 rpm. The supernatant was discarded, and the pellet was washed in 70% ethanol and dried at room temperature. The pellet was dissolved in 200 μL of TE (1X) buffer (1 mM Tris HCl pH 8.0, 0.1 mM EDTA pH 8.0). The eluted DNA was purified with PowerClean DNA Clean-up kit following manufacturer’s instructions (MoBio Laboratories, USA). After isolation, the quality and the size of the DNA was checked by electrophoresis using a 0.6% agarose gel and a 1Kb DNA marker (Takara, Japan), and the DNA concentration was quantified with a Qubit 2.0 Fluorometer (Invitrogen, Merelbeke, Belgium). To confirm that the specimen was *C. borealis*, we checked the ITS sequence with ITS1F/ITS4 primer^44^. The *C. borealis* genome was sequenced by DNALink Inc. using the PacBio RS II platform (Pacific Biosciences, USA). The PacBio SMRT bell library was long-read sequenced in five SMRT cells (Pacific Biosciences) using C4 chemistry (DNA Sequencing Reagent 4.0), and 1 × 240-min movies were captured for each SMRT cell. Illumina sequencing was used for correction during assembly: a paired-end (PE) library with an insert size of 350 bp was constructed in accordance with the manufacturer’s protocol and sequenced using the Illumina HiSeq platform (Illumina, Inc., San Diego, CA, USA).

Total RNA was extracted from the samples using the mirVana™ miRNA Isolation Kit (Ambion) following the manufacturer's recommended procedures. The purity was assessed on a NanoDrop8000 spectrophotometer with 1 μL of the total RNA extract. The integrity of the total RNA was evaluated using an Agilent Technologies 2100 Bioanalyzer, and the RNA Integrity Number (RIN) value was determined. The RNA-seq data was de novo assembled with Trinity (v2.15.0), and the Program to Assemble Spliced Alignments (PASA) annotation tool was used for gene predictions^45^.

### Genome assembly

De novo genome assembly was conducted using the PacBio long-read sequencing data with the FALCON-Unzip tool (v0.4)^46^. To enhance the assembly accuracy and correct errors in the draft *C. borealis* genome, Pilon (v1.22) was employed with the default parameters utilizing RNA-seq data generated from Illumina HiSeq^47^. To determine the completeness of the assembly, BUSCO (v4.1.2) was used with the Ascomycota_odb10 dataset^48^.

### Genome annotation

RepeatModeler (v2.0.1, RepeatModeler), with default parameters, was utilized to reveal transposable elements^49^. We used LTR_Retriever (v2.9.0) to identify long terminal repeat retrotransposons (LTR-RTs)^50^. The repeat elements were identified by constructing a de novo repeat library using RepeatMasker (v4.0.9)^51^.

Gene prediction was executed using PASA (v2.5.1)^52^. Followed by ab initio gene prediction using GeneMark (v4.2.9) and Augustus (v3.4.0) based on the repeat masked genome data^53,54^. Specifically, in GeneMark gene prediction, GeneMark-ES was utilized as the ab initio prediction in the first step. Through the ProtHint pipeline, hints were generated using fungi protein information from the ortho database, which we constructed for lichen annotation. Subsequently, protein equality alignment was performed in GeneMark-EP+^55^. Next, RNA-Seq data was used for PASA pipeline to provide evidence for the transcriptome data, which leads to more accurate genome prediction. All gene prediction data—including ab initio, transcriptome, and reference protein data—were integrated using EVidenceModeler (v1.1.1, EVM), which has been widely used for eukaryote gene prediction^52^. We reused PASA for the modification of the final gene models. Genome Annotation Generator (v2.0.1) was utilized to incorporate start and stop codons and generate a gff3 file for the genome annotation^56^.

### Functional annotation

Functional annotation was performed using BlastP (v2.2.29) based on a custom fungal database (E-value < 10^−5^)^57^. The custom fungal database was constructed by downloading the Fungi RefSeq database from the National Center for Biotechnology Information (NCBI). Protein domains were analyzed using InterProScan 5^58^. For the annotation of transcriptome assembly data, Trinotate (v3.2.0) was employed to provide comprehensive annotation information^59^. To decode the amino acid sequences, TransDecoder (v5.5.0) was utilized. Protein signal peptides were predicted using SignalP (v6.0)^60^. Additionally, protein family predictions were conducted using Pfam^61^. A GO analysis was conducted using Omicsbox software (v2.1.2)^62^.

### Comparative analysis within the genus *Cladonia*

For the comparative genomics analysis, we obtained the sampling locations and genomic sequences of five *Cladonia* species (*C. macilenta*^63^, *C. metacorallifera*^64^, *C. rangiferina*, *C. uncialis*, and *C. grayi*^65^) from NCBI and JGI which were isolated and sequenced from temperate climate (Table 3)^66^. The completeness of all genome assemblies was confirmed with BUSCO. To compare repeat element divergence between *Cladonia* species, we used LTR_retriever and RepeatMasker. We annotated four species (*C. grayi*, *C. macilenta*, *C. rangiferina*, and *C. uncialis*) with MAKER (v2.28) and *C. metacorallifera* was annotated on the GenSAS (v5.1, GenSAS, https://www.gensas.org) online platform^67^. For the *C. borealis* and *C. metacorallifera* genome alignment, MUMmer 4 was used^68^. Genome syntenies over 1 Mbp in length were visualized using Circos (v0.69-8)^69,70^.

To compare the six *Cladonia* species, OrthoFinder 2 was used with *Gomphillus americanus* protein sequences, downloaded from NCBI, as an outgroup^71^. *G. americanus* was used to ensure a meaningful comparison at the genus level, we chose species from different family that were registered in the annotation completed NCBI database. The orthologous gene families were gathered between the seven species based on their protein sequence similarities. Using single-copy ortholog data, evolutionary relationships were inferred by maximum-likelihood (ML) analysis using MEGA X (v10.2.4) with branch support assessed using 1000 bootstraps^72^.

### Prediction of BGCs and CAZymes

To explore secondary metabolite related BGCs, we searched our lichen genome sequences using antiSMASH fungal v7.0 with the default parameters^73^. CAZymes and CBM domains were annotated using run_dbcan 3 software with the default parameters by combining the search results of four databases: the EC number, HMMER, eCAMI, and DIAMOND databases^74^. To classify CAZyme families, we utilized the outcomes obtained from two or more databases.

### Expanded genes in *C. borealis*

Gene gain and loss were analyzed using CAFÉ 4.0 with gene family expansions or contractions considered significant at *P* < 0.05^75^. A Venn diagram was drawn using the Orthovenn 2 web server to visualize orthologous gene families among *Cladonia* species^76^. Based on the CAFÉ analysis result, *C. borealis*-specific genes were identified. We manually annotated expanded genes in *C. borealis* and the five other *Cladonia* species using four databases—including NCBI reference sequences, Uniprot reviewed sequences, GO terms, and Pfam—based on a BlastP E-value cut-off of 1e−12 and a homology of 50% or above^77^. Among transporter proteins, we sorted transmembrane transporters and classified genes by function. Annotated sequences were once again confirmed with the NCBI Conserved Domain Database (CDD) database. The evolutionary relationships between *Cladonia* species, based on the expanded or contracted protein families were analyzed through ML inference using MEGA X with branch support based on 1000 bootstrap sample trees^72^.

### Supplementary Information

Supplementary Information.

## Data Availability

The dataset utilized in this research can be accessed through an online repository. The name of the repository and accession number are BioProject ID; PRJNA693578. This whole genome shotgun project has been deposited at DDBJ/ENA/GenBank under the accession number JAFEKC000000000. The version described in this paper is version JAFEKC020000000.
