# Supplementary material for: An Antarctic lichen isolate (Cladonia borealis) genome reveals potential adaptation to extreme environments
Source: Sci Rep. 2024 Jan 16;14:1342. doi: 10.1038/s41598-024-51895-x (PMC10792129; doi:10.1038/s41598-024-51895-x)
Supplement: Supplementary file 1 — Supplementary Information. [file 41598_2024_51895_MOESM1_ESM.pdf]

Supple data

Region	No. of Bases	No. of bases		No. of reads		Mean subread length (bp)
		(bp)	(%)	(bp)	(%)	
~ 2kb	18,654,189	93,270,946	1.89	83,090	13.07	1,123
~3kb	25,967,447	129,837,235	2.62	53,394	8.40	2,432
~4kb	30,703,163	153,515,816	3.10	43,866	6.90	3,500
~5kb	39,933,916	199,669,580	4.04	44,376	6.98	4,499
~6kb	48,622,995	243,114,977	4.91	44,219	6.95	5,498
~7kb	56,210,394	281,051,971	5.68	43,249	6.80	6,498
~8kb	60,895,826	304,479,129	6.16	40,618	6.39	7,496
~9kb	64,808,988	324,044,939	6.55	38,148	6.00	8,494
~10kb	69,206,091	346,030,453	7.00	36,449	5.73	9,494
10kb~	574,347,78	2,871,738,916	58.05	208,534	32.79	13,771
Total	**989,350,792**	**4,946,753,962**	**100**	**635,943**	**100**	**7,779**

Table S1. Sequencing results summary of *Cladonia borealis*.

	Repeat elements	Repeat length (bp)					
		**Cbo***[*]	**Cuc**	**Cgr**	**Cma**	**Cme**	**Crg**
DNA							21 (2,362)
	CMC-EnSpm	39 (6,739)			66 (50,963)		
	Crypton-F						13 (8,520)
	IS3EU						6 (17,400)
	Kolobok-H	45 (95,598)					
	PIF-Harbinger	110 (14,359)					
	TcMar-Ant1					34 (24,124)	
	TcMar-Fot1	15 (7,028)				80 (101,358)	
	hAT-Ac					56 (23,213)	162 (29,816)
	hAT-Charlie	61 (30,236)	7 (1,662)		162 (37,007)		12 (6,045)
	Maverick		128 (19,579)			194 (49,257)	
	MULE-MuDR			20 (8,342)		43 (19,597)	
LINE	L1	29 (3,716)		31 (11,327)			
	Penelope						29 (32,813)
LTR	Copia	392 (723,281)	155 (197,176)	182 (401,624)	324 (305,125)	231 (231,384)	592 (562,274)
	ERVK	271 (36,087)					
	Gypsy	875 (1,366,219)	274 (334,280)	940 (1,757,486)	882 (1,640,788)	1,148 (1,941,677)	842 (1,348,871)
	Pao	14 (1,816)					
	Unknown		104 (36,237)	214 (93,982)	265 (287,491)	5 (702)	544 (292,897)
Unknown		4,197 (1,055,932)	3,685 (1,024,240)	3,476 (1,408,875)	5,017 (2,155,669)	4,711 (1,445,732)	5,044 (1,399,553)
Low complexity		794 (45,137)	552 (29,811)	783 (45,029)	838 (47,910)	905 (53,859)	772 (42,313)
Satellite				29 (7,006)			
Simple repeat		4,806 (245,821)	3,527 (163,417)	4,306 (205,055)	4,133 (198,755)	4,662 (235,251)	3,850 (180,960)
rRNA		29 (91,060)					
**Total**		**11,677 (3,723,029)**	**8,432 (1,806,402)**	**9,981 (3,938,726)**	**11,687 (4,723,708)**	**12,069 (4,126,154)**	**11,887 (3,923,824)**

*See Table 1 for abbreviations

Table S2. Identification of repeat elements in *Cladonia* species genome.

Abbr.	Number of scaffolds	Total size of scaffolds (bp)	Longest scaffold (bp)	Scaffold N50 (bp)	BUSCO complete	Gene number	Annotation method
Cbo*	48	36,013,978	2,231,325	1,657,173	93.9%	10,749	EVM
Cgr	414	34,622,149	958,967	243,412	97.4%	10,248	Maker
Cma	240	37,117,081	2,265,542	1,469,036	97.7%	8,276	Maker
Cme	30	36,682,060	2,400,105	1,591,850	96.3%	10,825	GenSAS
Crg	1,069	35,673,945	751,829	273,041	98.0%	8,568	Maker
Cuc	2,124	32,851,544	143,175	34,871	93.3%	8,404	Maker
Gam	54	23,861,785	1,851,112	685,684	95.7%	8,300	Maker

*See Table 1 for abbreviations

Table S3. Assembly information of species genomes.

Sequence	Scaffold			Length (aa)	Forward/Reverse
	Location	Start	End		
JMJ3500000884-RA	scf7180000000116	669768	671876	712	+
JMJ3500001901-RA	scf7180000000117	1385261	1389721	1507	+
JMJ3500001983-RA	scf7180000000117	1629904	1634397	1328	-
JMJ3500002017-RA	scf7180000000117	1721321	1726670	1618	+
JMJ3500000709-RA	scf7180000000118	2159401	2164553	1485	-
JMJ3500002664-RA	scf7180000000119	1593316	1598077	1553	-
JMJ3500003346-RA	scf7180000000120	1678279	1683080	1601	+
JMJ3500006068-RA	scf7180000000121	57704	62417	1407	+
JMJ3500006095-RA	scf7180000000121	142243	144494	744	-
JMJ3500007183-RA	scf7180000000123	413644	417149	1070	+
JMJ3500004058-RA	scf7180000000124	442727	447583	1605	-
JMJ3500004126-RA	scf7180000000124	673246	678416	1727	-
JMJ3500004215-RA	scf7180000000124	941402	945541	1364	+
JMJ3500004257-RA	scf7180000000124	1044729	1049489	1571	-
JMJ3500003445-RA	scf7180000000125	150373	152408	649	+
JMJ3500007709-RA	scf7180000000130	501179	506071	1653	-
JMJ3500005630-RA	scf7180000000134	185259	187773	831	-
JMJ3500005853-RA	scf7180000000134	925837	928752	949	-
JMJ3500009919-RA	scf7180000000139	1055440	1060470	1638	+

*See Table 1 for abbreviations

Table S4. ABC transport protein's characterization in *C. borealis* genome. The gene direction represented in (+) for forward, (-) for reverse.

Sequence	Scaffold			Length (aa)	Forward/Reverse
	Location	Start	End		
JMJ3500000716-RA	scf7180000000118	2185425	2186303	296	-
JMJ3500002260-RA	scf7180000000119	291180	293395	727	-
JMJ3500002578-RA	scf7180000000119	1296785	1299605	894	-
JMJ3500002883-RA	scf7180000000120	287061	287786	208	-
JMJ3500010396-RA	scf7180000000122	311726	313455	461	+
JMJ3500010453-RA	scf7180000000122	516292	517684	435	-
JMJ3500007066-RA	scf7180000000123	75792	77782	585	-
JMJ3500007326-RA	scf7180000000123	1018149	1020027	600	+
JMJ3500003990-RA	scf7180000000124	194311	196077	559	-
JMJ3500004385-RA	scf7180000000124	1457511	1459517	677	+
JMJ3500010234-RA	scf7180000000127	266624	267915	399	+
JMJ3500008012-RA	scf7180000000129	136982	139359	766	+
JMJ3500008057-RA	scf7180000000129	257739	258788	316	-
JMJ3500008282-RA	scf7180000000129	1013906	1015886	599	+
JMJ3500006012-RA	scf7180000000134	1401601	1403833	645	-
JMJ3500010004-RA	scf7180000000138	192004	195817	1074	+

*See Table 1 for abbreviations

Table S5. Magnesium transport protein's characterization in *C. borealis* genome. The gene direction represented in (+) for forward, (-) for reverse.

**CAZyme family**	**Cbo***	**Cgr**	**Cma**	**Cme**	**Crg**	**Cuc**
AA1	8	10	9	10	14	11
AA3	1	1	0	0	1	2
AA5+CBM32	1	0	2	2	0	2
CBM1+GH5	1	1	1	1	1	1
CBM18+GH16	1	1	1	1	0	1
CBM18+GH18	0	1	0	0	0	0
CBM2+GH18	0	0	0	0	0	1
CBM20+GH15	1	1	1	1	1	1
CBM24+GH71	1	1	1	1	1	1
CBM32+AA5	1	1	0	0	2	0
CBM43+GH72	1	1	1	1	1	1
CBM48+GH13	1	1	1	1	1	1
GH125	1	1	1	1	1	1
GH13	2	1	1	2	2	1
GH13+GT5	2	2	2	2	2	2
GH132	1	0	1	1	1	1
GH133+GH13	1	1	1	1	1	1
GH152	0	0	1	1	0	0
GH16	3	3	2	2	2	3
GH17	2	1	2	2	2	1
GH18	2	2	1	1	1	1
GH2	3	3	3	3	3	3
GH20	1	1	1	1	1	1
GH25	1	1	1	1	1	1
GH27	1	1	1	1	1	1
GH3	4	4	4	4	4	4
GH31	3	3	2	2	3	3
GH32	1	1	1	1	1	1
GH35	1	1	1	1	1	1
GH37	2	1	2	2	2	2
GH38	1	1	1	1	1	1
GH47	4	4	4	4	4	4
GH5	6	6	5	6	7	6
GH55	2	2	2	2	2	2
GH63	1	1	1	1	1	1
GH72	2	2	2	2	2	2
GH81	1	0	1	1	1	1

*See Table 1 for abbreviations

Table S6. Number of CAZyme family's distribution in *Cladonia* species.

CAZyme family	Cbo*	Cgr	Cma	Cme	Crg	Cuc
GT1	3	3	3	3	3	3
GT15	2	2	2	2	2	2
GT2	10	9	9	10	9	9
GT20	1	1	1	1	1	1
GT21	1	1	1	1	1	1
GT22	2	2	1	1	2	0
GT24	1	1	1	1	1	1
GT3	1	1	1	1	1	1
GT32	2	2	2	2	2	1
GT33	1	0	0	1	0	1
GT34	1	1	1	1	1	1
GT35	1	1	1	1	1	1
GT39	3	3	2	3	3	3
GT4	2	3	3	3	3	3
GT48	1	1	1	1	1	1
GT57	2	2	2	2	2	2
GT58	1	1	0	0	1	1
GT69	1	2	1	1	1	1
GT8	1	1	1	1	1	1
**Total**	**102**	**99**	**94**	**100**	**105**	**101**

*See Table 1 for abbreviations

Table S6. *Cont.*

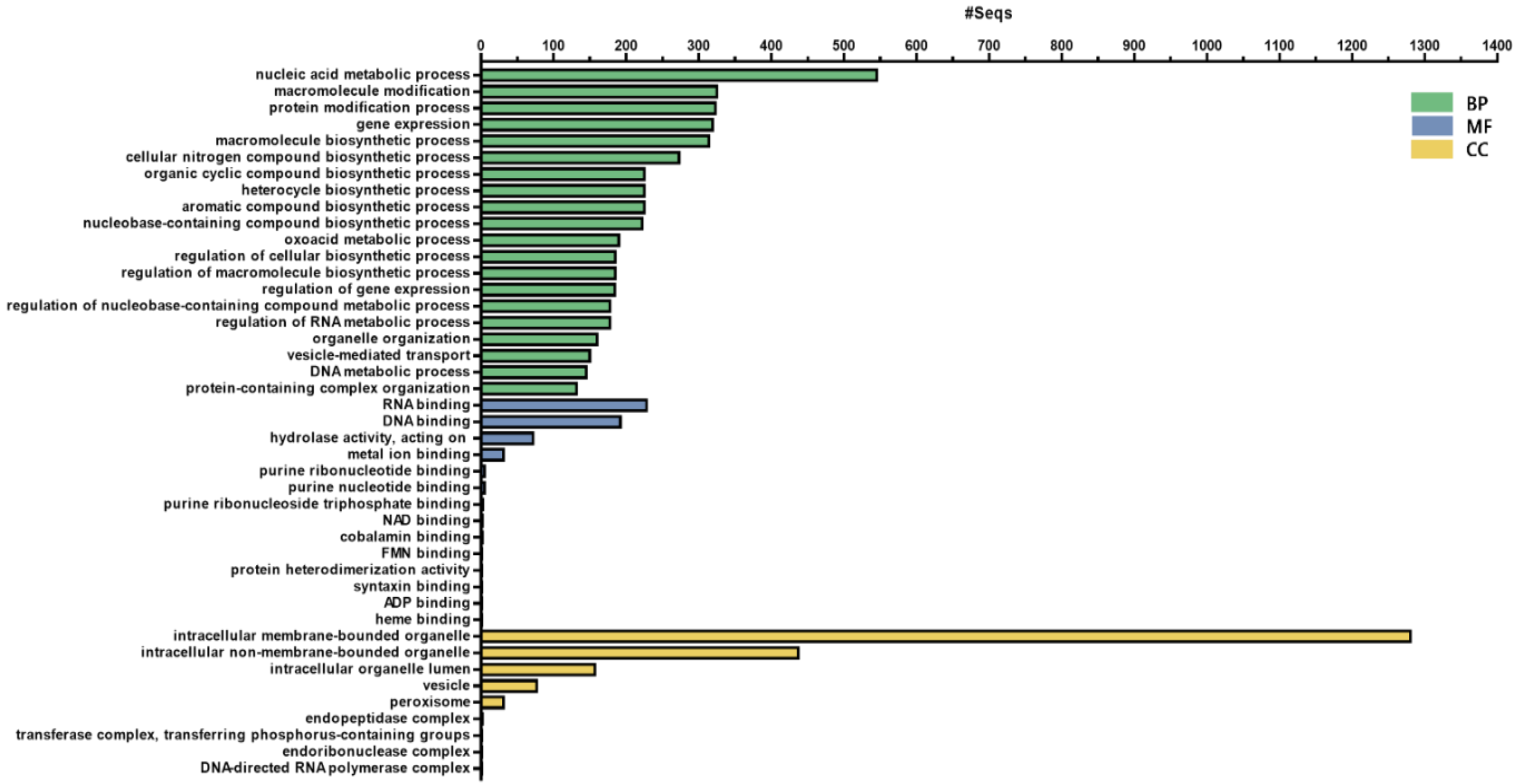

Figure S1. Distribution of top20 GO terms in level 5 GO functional classification by using Omicsbox. Green, blue, and yellow colors represent biological process (BP), molecular function (MF), and cellular component (CC), respectively.

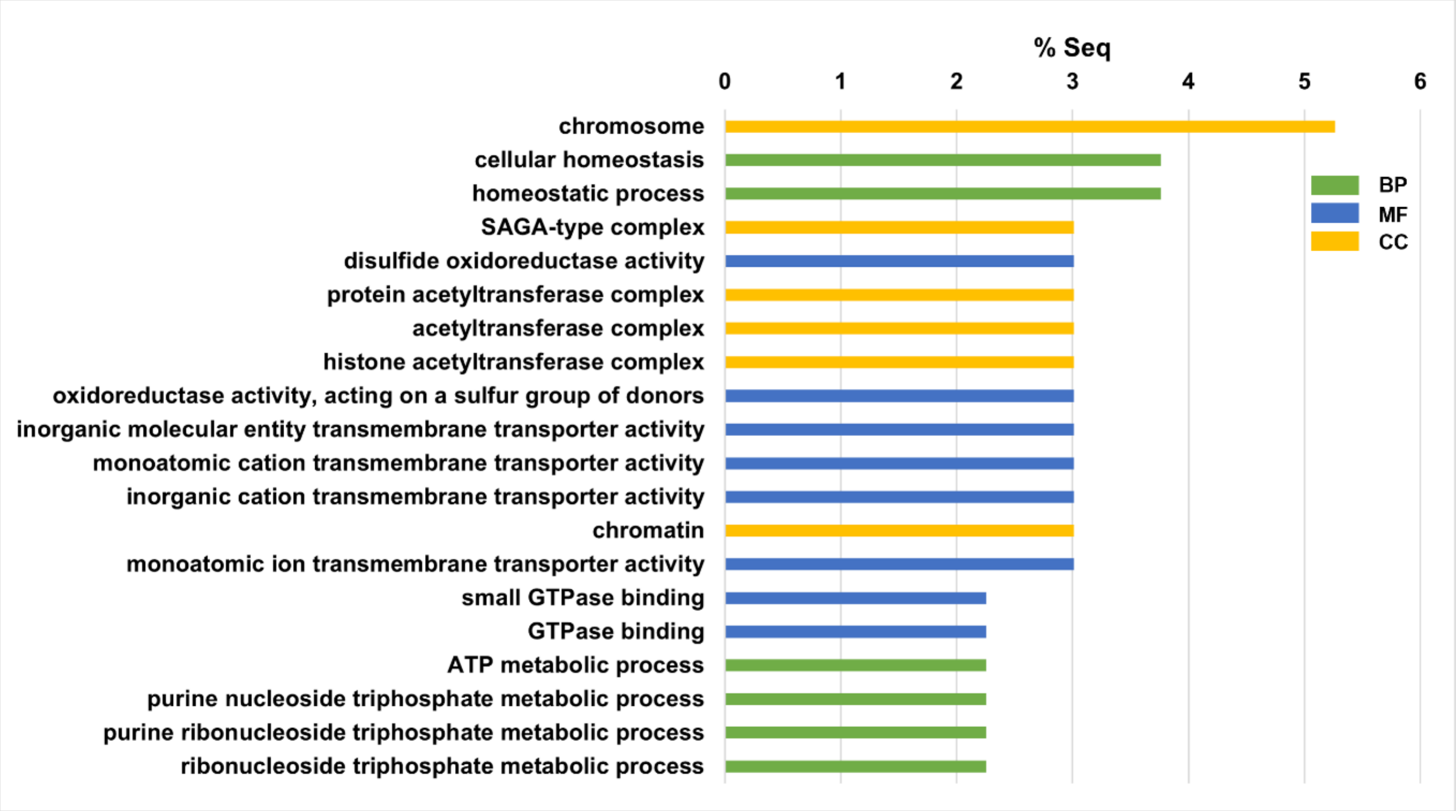

Figure S2. GO enriched bars represent top 20 expanded gene families in *C. borealis* genome among six *Cladonia* species.
